# Surgical Treatment of Peritoneal Carcinomatosis from Gastric Cancer

**DOI:** 10.1155/2012/405652

**Published:** 2012-06-20

**Authors:** Kiran K. Turaga, T. Clark Gamblin, Sam Pappas

**Affiliations:** Division of Surgical Oncology, Medical College of Wisconsin, Milwaukee, WI 53226, USA

## Abstract

Peritoneal carcinomatosis from gastric cancer is considered a fatal disease with limited treatment options. Recent advances in the understanding of the disease process, systemic chemotherapy, and application of cytoreductive surgery and hyperthermic chemoperfusion have shown promising results in the management of this difficult disease. Novel therapies such as extensive intraperitoneal lavage and intraperitoneal targeted agents are being applied in the management of this disease. We review the current literature in this field and describe the rationale behind some of these advances.

## 1. Introduction

Gastric cancer is the second leading cause for cancer-related mortality worldwide with almost 22,280 patients being diagnosed with gastric cancer annually in the United States [1, 2]. Peritoneal dissemination occurs commonly in patients with gastric cancer by means of intracoelomic dissemination or due to tumor spillage at the time of an operation [3]. High risk of peritoneal carcinomatosis from gastric cancer has led to common use of laparoscopy in the management of patients with gastric cancer. Unfortunately, systemic chemotherapy has not been shown to have a significant benefit to patients with peritoneal carcinomatosis. Despite short-duration response rates (43%) for visceral metastases with epirubicin-, cisplatin-, and 5-fluorouracil-based regimens, the response rate for peritoneal carcinomatosis is less than 14% [3, 4]. The blood peritoneal barrier which is 90 *μ*m wide prevents a high concentration of intravenous chemotherapy from accumulating in the peritoneal surface, and this has led to increased interest in locoregional treatment for peritoneal carcinomatosis (PC) from gastric cancer [3].

Surgery has been the mainstay for treatment of gastric cancer without peritoneal dissemination, but advances in neoadjuvant chemotherapy with the MAGIC trial have led to significant survival benefits for patients [5]. This strategy demonstrated an effective use of multimodality therapy for patients with gastric cancer. 

The application of hyperthermic intraperitoneal chemoperfusion or HIPEC to gastric cancer has been described by several groups over the last decade [3, 6, 7]. This technique refers to the combination of extensive cytoreductive surgery performed by an experienced team to remove all visible tumor, followed by intraoperative circulation of heated chemotherapy in the abdominal cavity during the procedure. This technique is currently considered standard of care for patients with PC from colorectal cancer, pseudomyxoma peritonei, and mesotheliomas [8]. 

Despite being described in the late 20th century, and its attractive method of delivery of chemotherapy to bypass the blood-peritoneal barrier, the adoption of HIPEC for the treatment of gastrointestinal malignancies was not common until 2003 when a randomized controlled trial demonstrated a doubling of survival for patients with PC from colorectal cancer [9]. The application of HIPEC to patients with gastric cancer was reported by several western groups at the same time, which also showed promising results [6].

The rationale behind cytoreductive surgery for regional spread of disease includes removal of all peritoneal surfaces bearing tumor by using traditional surgery in combination with techniques including electrosurgery. Direct application of chemotherapy agents to the peritoneal surfaces in combination with hyperthermia (approximately 42°celsius) allows for direct tissue penetration of the chemotherapy, with limited systemic side effects.

We describe in our paper the application of HIPEC to the treatment of patients with gastric cancer and provide an evidence-based review of outcomes of patients undergoing regional therapies for PC.

## 2. History of HIPEC for Gastric Cancer

The use of intraperitoneal therapy for the management of malignant ascites was described as early as the 18th century when wine and Bristol water were instilled in the peritoneal cavity [10]. Since then, numerous advances have been made in the intraperitoneal management of tumors with regional spread. In fact, the NCI recognized intraperitoneal therapy as the standard of care in 2006 for ovarian cancer based on numerous randomized controlled trials [7]. Randomized data also supports the use of hyperthermic intraperitoneal chemoperfusion for patients with colorectal cancer with PC. 

The application of HIPEC to patients with advanced gastric cancer was reported in 1988 by Fujimoto et al. who reported a median survival of 7.2 months in 15 patients [11]. Subsequently, several western centers adopted this technique with reports of 1-year survival of 43–45% [6, 12]. A meta-analysis reported by Yan et al. in 2007 identified randomized trials performed from 1983 to 2002, and a pooled analysis of the trials using hyperthermic intraperitoneal therapy showed a survival benefit with a pooled hazards ratio of 0.60 (95% CI 0.43–0.83, *P* = 0.002) when compared to surgery alone [7]. This survival benefit was improved significantly when early postoperative intraperitoneal chemotherapy was added to the HIPEC arm (HR 0.45 (95% CI 0.29–0.68, *P* = 0.0002)) [7]. 

## 3. Recent Randomized Trials

Recent randomized trials have shown very promising results for patients with PC from gastric cancer, and these reflect not only the advancements in surgery and anesthesia but also multimodality therapy being applied to these patients. Yang et al. reported a randomized controlled trial with 68 patients, with 34 in each arm who were randomized to get CRS+HIPEC versus CRS alone [13]. This trial was not stratified on the peritoneal carcinomatosis index (PCI) which is a marker of the burden of disease in the peritoneal cavity and consequentially complete cytoreduction was achieved only in 58.8% in both arms. Despite this, the median survival was increased by 70% in the CRS+HIPEC arm with a median survival of 11.5 months. This was in concordance with papers published in the US, and Europe previously [3].

Despite showing a significant benefit in survival, the overall survival remains short. Nevertheless, novel applications of intraperitoneal therapy seem to offer patients the most effective therapy to date. Kuramoto and colleagues reported the use of extensive intraoperative peritoneal lavage in conjunction with intraperitoneal chemotherapy for patients with cytology only positive gastric cancer who underwent surgical resection [14]. These investigators reported the use of 10 liters of lavage after surgical resection in conjunction with intraperitoneal chemotherapy for patients with gastric cancer without evidence of tumor deposits. In this randomized trial, the investigators reported a 5-year survival of 43% which exceeds previous papers for gastric cancer. In this trial, the authors did not report an increased incidence of adverse effects from the extensive lavage, although the adverse effects themselves were not reported. It is possible that the lavage can lead to dyselectrolytemia and increased GI toxicity; however, this needs to be studied further.

Papers such as those from Kuramoto et al. [14] have led investigators to believe that the application of HIPEC for advanced gastric cancer is aimed to prevent peritoneal carcinomatosis or to act when the burden of disease is extremely low. Glehen et al. have found that patients with a low burden of disease (peritoneal carcinomatosis index <9) have a significantly better survival after complete cytoreductive surgery and hyperthermic chemoperfusion [12]. This has also led Yonemura et al. to propose bidirectional neoadjuvant chemotherapy in which patients with low-volume peritoneal disease are given neoadjuvant intravenous (IV) and intraperitoneal chemotherapy and then taken to the operating room for cytoreductive surgery and hyperthermic chemoperfusion [3].

Currently, the EUNE protocol (European Union network of excellence on gastric cancer) includes patients with aggressive histology but low-volume peritoneal disease such as T3-T4 lesions with node-positive disease or patients with positive peritoneal cytology [15]. All patients will receive three cycles of IV platinum-based therapy similar to the MAGIC protocol, followed by D2 surgical resection. Patients will then be randomized to undergo HIPEC with oxaliplatin versus the surgical resection alone. The trial is aimed to demonstrate the efficacy of regional therapies in both prevention of recurrence and overall survival.

## 4. Approach to Advanced Unresectable Peritoneal Disease

**Figure 1:**
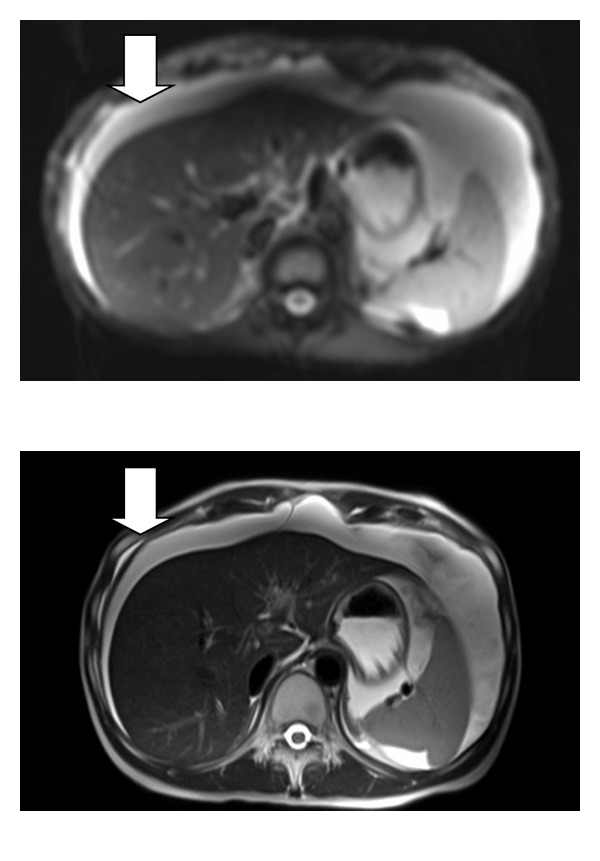
Diffusion-weighted MRI demonstrating ascites (arrow) in patients with gastric cancer following partial gastrectomy with chemotherapy-associated steatohepatitis.

**Figure 2:**
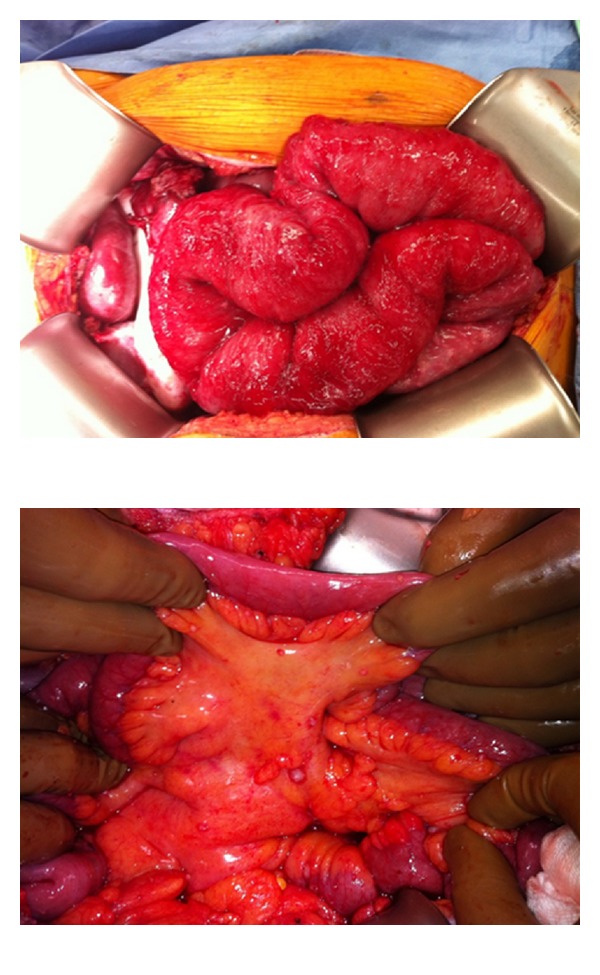
Mesenteric disease from gastric cancer primary leading to bowel obstruction.

Patients with peritoneal carcinomatosis often develop significant bowel obstructions, intractable ascites, and cachexia (Figures 1 and 2). Surgical approaches for best symptom palliation should be widely used at centers of peritoneal surface malignancy. Surgical bypasses or venting gastrostomy tubes even in the setting of gastric cancer are acceptable for palliation. The application of intraperitoneal therapy such as anti-EPCAM (epithelial cell adhesion) antibodies has shown significant benefits in puncture-free survival (survival without repeated paracentesis) for patients with malignant ascites in a phase II/III randomized trial [16]. In addition, for EPCAM+ tumors, the use of intraperitoneal catumaxomab has also shown an improved progression-free interval in phase II studies [17]. The use of catumaxomab in the United States has been restricted due to the pending FDA approval. Catumaxomab was fairly well tolerated in the published trial with pyrexia and abdominal pain being the most common side effects (60% and 43%, resp.). However, the incidence of individual grade III toxicities was <10%. 

## 5. Conclusions

Patients with peritoneal carcinomatosis from gastric cancer have novel surgical options for treatment of disease. The approach is justified by data reported, and selection is of paramount importance. The application of cytoreductive surgery and hyperthermic chemoperfusion appears most favorable for patients with low-volume disease. Use of techniques such as extensive peritoneal lavage and intraperitoneal anti-EPCAM antibodies (i.e., catumaxomab) is an exciting advance in the treatment of these patients. Surgical palliation must be considered for patients who have intractable symptoms from the disease.
